# 

**Published:** 1886-06

**Authors:** 


### AMERICA.

### ARGENTINE REPUBLIC.

*Monthly.*

*Anales del Circulo Médico Argentino.* Buenos Ayres: M. Biedma.

### CANADA.

*Monthly.*

*Canada Medical and Surgical Journal.* Montreal: Gazette Printing Company.

### PERU.

*Monthly.*

*La Crónica Médica.* Lima: Carlos Prince.

### UNITED STATES.

*Weekly.*

*Technics.* Boston: 51 Union Park.

*The Cincinnati Lancet-Clinic.* Cincinnati: 281 West 7th Street.

*The Journal of the American Medical Association.* Chicago: 65 Randolph Street.

*The Medical and Surgical Reporter.* Philadelphia: 115 South Seventh Street.

*The Medical Record.* New York: William Wood & Co.

*The New York Medical Journal.* New York: D. Appleton & Co.

*Fortnightly.*

*Philadelphia Medical Times.* Philadelphia: J. B. Lippincott & Co.

*The American Practitioner & News.* Louisville: John P. Morton and Company.

*Monthly.*

*Index Medicus.* Detroit: G. S. Davis.

*Journal of Cutaneous and Venereal Diseases.* New York: William Wood & Company.

*Pacific Medical and Surgical Journal and Western Lancet.* San Francisco: W. S. Duncombe.

*The American Journal of Ophthalmology.* St. Louis: J. H. Chambers & Co.

*The Medical Analectic.* New York: G. P. Putnam's Sons.

*The Obstetric Gazette.* Cincinnati: 281 West 7th Street.

*The Polyclinic.* Philadelphia: P. Blakiston, Son & Co.

*The Saint Louis Medical and Surgical Journal.* Saint Louis: 2622 Washington Avenue.

*The Therapeutic Gazette.* Detroit: George S. Davis.

*Quarterly.*

*The American Journal of the Medical Sciences.* Philadelphia: Lea Brothers & Co.

*Yearly.*

*Transactions of the American Surgical Association.* Philadelphia: P. Blakiston, Son & Co.

*Occasionally.*

*Transactions of the New York Academy of Medicine.* New York: 12 West 31st Street.

### ASIA.

### INDIA.

*Monthly.*

*The Indian Medical Journal.* Calcutta: W. Newman & Co., Ld.

### JAPAN.

*Monthly.*

*The Sei-I-Kwai Medical Journal.* Tokio: Z P. Maruya & Co.

### AUSTRALASIA.

### AUSTRALIA.

*Monthly.*

*The Australasian Medical Gazette.* Sydney: L. Bruck.

*The Australian Medical Journal.* Melbourne: Stillwell & Co.

### EUROPE.

### BELGIUM.

*Monthly.*

*Bulletin de l'Académie royale de médecine de Belgique.* Brussels: A. Manceaux.

### BRITISH ISLES.

*Weekly.*

*British Medical Journal.* London: 161A Strand.

*The Lancet.* London: 423 Strand.

*Monthly.*

*Annals of Surgery.* London: Baillière, Tindall & Cox.

*The Birmingham Medical Review.* Birmingham: Cornish Bros.

*The London Medical Record.* London: Smith, Elder, & Co.

*The Medical Chronicle.* Manchester: John Heywood.

*The Practitioner.* London: Macmillan & Co.

*Edinburgh Medical Journal.* Edinburgh: Oliver and Boyd.

*The Glasgow Medical Journal.* Glasgow: Alex. Macdougall.

*The Dublin Journal of Medical Science.* Dublin: Fannin & Co.

*Quarterly.*

*The Asclepiad.* London: Longmans, Green, & Co.

*The British Gynæcological Journal.* London: Smith, Elder, & Co.

*Half-yearly.*

*The Liverpool Medico-Chirurgical Journal.* Liverpool: Medical Institution.

*The Retrospect of Medicine.* London: Simpkin, Marshall, & Co.

*Yearly.*

*The Medical Annual.* London: Henry Kimpton.

*The Year-Book of Treatment.* London: Cassell & Company, Limited.

*Transactions of the Academy of Medicine in Ireland.* Dublin: Fannin and Company.

DENMARK.

*Weekly.*

*Hospitals-Tidende.* Copenhagen: Jacob Lund.

FRANCE.

*Three times a week.*

*Gazette des hôpitaux.* Paris: 4 Rue de l'Odéon.

*L'Union médicale.* Paris: 11 Rue de la Grange-Batelière.

*Weekly.*

*Bulletin de l'Académie de médecine.* Paris: G. Masson.

*Gazette médicale de Paris.* Paris: 8 Place de l'Odéon.

*Le Progrès médical.* Paris: 14 Rue des Carmes.

*Monthly.*

*Gazette de Gynécologie.* Paris: O. Doin.

*Nouvelles Archives d'Obstétrique et de Gynécologie.* Paris: Félix Alcan.

*Revue mensuelle de Laryngologie, d'Otologie et de Rhinologie.* Paris: 8 Place de l'Odéon.

GERMANY.

*Twice a week.*

*Deutsche Medizinal-Zeitung.* Berlin: Eugen Grosser.

*Weekly.*

*Medicinische Bibliographie.* Leipzig: Breitkopf & Härtel.

GREECE.

*Weekly.*

Γαληνὸς. Athens: N. G. Passare.

HOLLAND.

*Weekly.*

*Nederlandsch Tijdschrift voor Geneeskunde.* Amsterdam: F. van Rossen.

ITALY.

*Monthly.*

*Lo Sperimentale.* Florence: 35 Via degli Alfani.

NORWAY.

*Monthly.*

*Norsk Magazin for Lægevidenskaben.* Christiania: Th. Steens.

PORTUGAL.

*Weekly.*

*A Medicina Contemporanea.* Lisbon: José Antonio Rodrigues & Cª

ROUMANIA.

*Monthly.*

*Spitalul.* Bucharest: Stefan Mihalescu.

RUSSIA.

*Weekly.*

*Vrach.* St. Petersburg: C. Ricker.

SPAIN.

*Weekly.*

*El Siglo Médico.* Madrid: Magdalena, 36, 2º

SWEDEN.

*Quarterly.*

*Nordiskt Medicinskt Arkiv.* Stockholm: P. A. Norstedt & Söner.

SWITZERLAND.

*Monthly.*

*Illustrirte Monatsschrift der ärztlichen Polytechnik und Centralblatt der orthopädischen Chirurgie.* Berne: Schmid, Francke & Co.

### PUBLICATIONS RECEIVED.

From Trow's Printing and Bookbinding Company, New York:
*Comments on Pasteur's Method of Treating Hydrophobia.* By Charles W. Dulles, M.D. 1886. Reprint.

From Bigelow Brothers, Buffalo, N.Y.:
*The Medical Press.* Vol. I. Nos. 6 and 7.

From John P. Morton and Company, Louisville, Ky.:
*Esthetics of Medicine.* By H. A. Cottell, M.D. 1886. Reprint.

From Cassell and Company, Limited, London:
*A Manual of Surgery.* Edited by Frederick Treves. 3 vols. 1886.
*Materia Medica and Therapeutics.* By J. Mitchell Bruce, M.A., M.D. Third Edition. 1886.

From Young J. Pentland, Edinburgh:
*Atlas of Venereal Diseases.* Maclaren. Fasc. VI.
*Medical Tracts for the Times.* By J. Craig and W. Maule.
*Synopsis of Therapeutics.* By R. S. Aitchison.

From J. and A. Churchill, London:
*On Inversion of the Uterus.* By J. H. Aveling.

Bristol Medico-Chirurgical Journal.

JUNE, 1886.

*Cases for binding, price 7d. each, may be had of the Publisher,*
J. W. ARROWSMITH, *Quay Street, Bristol.*

### Bristol Medico-Chirurgical Society.

---

**President.**
W. H. SPENCER, M.D., M.A. Cantab.

**President-Elect.**

F. POOLE LANSDOWN.

**Honorary Secretary.**
R. SHINGLETON SMITH, M.D., B.Sc. Lond

**Committee.**

*Past Presidents.*
R. W. COE.
AUGUSTIN PRICHARD, M.D. Berlin.
J. G. SWAYNE, M.D. Lond.
E. LONG FOX, M.D. Oxon.
JAMES G. DAVEY, M.D. St. And.
W. JOHNSTONE FYFFE, M.D. Dub.
G. F. BURDER, M.D., Aberd.

F. RICHARDSON CROSS, M.B. Lond.
NELSON C. DOBSON.
L. M. GRIFFITHS.
A. J. HARRISON, M.B. Lond.
E. MARKHAM SKERRITT, M.D., B.S., B.A. Lond.
J. GREIG SMITH, M.B., C.M., M.A. Aberd., F.R.S.E.

Medical Men wishing to join the Society are requested to communicate with the Honorary Secretary, Dr. R. Shingleton Smith, 9 Richmond Hill, Clifton. The Annual Subscription is 10/6.

---

*Extracts from Journal By-laws.*

4.—The *Journal* shall be delivered free to Subscribers and also to Members of the Society whose subscriptions are not in arrear.

6.—The Annual Subscription to the *Journal* for those who pay before the 1st of July shall be 5/-, post free, or 6/- if paid after that date.

---

Communications intended for insertion in the *Journal*, Books for Review, &c., should be sent to the Editor, J. Greig Smith, M.A., F.R.S.E., 16 Victoria Square, Clifton.

Exchange Journals and communications referring to the delivery of the *Journal* should be sent to the Assistant-Editor, L. M. Griffiths, 9 Gordon Road, Clifton, to whom Subscriptions are to be paid *in advance*.

Authors requiring Reprints of their Papers should communicate with the Publisher.

Cases for Binding Vols. I., II., and III. are now ready, and may be obtained, price 7*d.* each (postage 2*d.* extra), upon application to J. W. Arrowsmith, Quay Street, Bristol; or the *Journal* will be bound, including Case, for 1/3 per volume.

